# The role of *cydB* gene in the biofilm formation by *Campylobacter jejuni*

**DOI:** 10.21203/rs.3.rs-4342718/v1

**Published:** 2024-09-10

**Authors:** Jakub Korkus, Patrycja Sałata, Stuart A. Thompson, Emil Paluch, Jacek Bania, Ewa Wałecka-Zacharska

**Affiliations:** 1 Department of Food Hygiene and Consumer Health Protection, Wrocław University of Environmental and Life Sciences, Wrocław, Poland; 2 Division of Infectious Diseases, Department of Medicine, Medical College of Georgia Augusta University, Augusta, GA USA; 3 Department of Microbiology, Faculty of Medicine, Wroclaw Medical University, Wroclaw, Poland

**Keywords:** *C. jejuni*, biofilm, transposon mutagenesis, *cydB*

## Abstract

*Campylobacter jejuni* is a major cause of food- and water-borne bacterial infections in humans. A key factor helping bacteria to survive adverse environmental conditions is biofilm formation ability. Nonetheless, the molecular basis underlying biofilm formation by *C. jejuni* remains poorly understood. Around thirty genes involved in the regulation and dynamics of *C. jejuni* biofilm formation have been described so far. We applied random transposon mutagenesis to identify new biofilm-associated genes in *C. jejuni* strain 81–176. Of 1350 mutants, twenty-four had a decreased ability to produce biofilm compared to the wild-type strain. Some mutants contained insertions in genes previously reported to affect the biofilm formation process. The majority of identified genes encoded hypothetical proteins. In the library of EZ-Tn5 insertion mutants, we found the *cydB* gene associated with respiration that was not previously linked with biofilm formation in *Campylobacter*. To study the involvement of the *cydB* gene in biofilm formation, we constructed a non-marked deletion *cydB* mutant together with a complemented mutant. We found that the *cydB* deletion-mutant formed a weaker biofilm of loosely organized structure and lower volume than the parent strain.

In the present study, we demonstrated the role of the *cydB* gene in biofilm formation by *C. jejuni*.

## Introduction

*Campylobacter jejuni* is a major cause of human food- and water-borne bacterial infections. In the European Union, campylobacteriosis is the most prevalent zoonosis and has been so since 2005 [^1^]. The primary source of human infections are undercooked poultry meat and poultry products, although cross-contamination of fresh produce and cooking utensils is also a possible transmission route [^2^]. Campylobacteriosis is usually manifested by fever, severe abdominal pain, and diarrhea. In a minority of patients, postinfectious sequellae may occur, including Miller-Fisher or Guillain-Barré syndromes [^3^]. The bacterium cannot grow under 30°C, requires a reduced oxygen atmosphere, and is sensitive to desiccation, high/low temperatures, osmotic and acid stress. The survival mechanism of Campylobacter in the natural and food processing environment is not clear. Scientists have postulated that biofilm formation is a relevant survival strategy under unpropitious conditions. Biofilm is a community of surface-attached microorganisms of one or more species enclosed in a self-produced extracellular matrix. Extracellular matrix composition varies by species but typically consists of DNA, proteins, and extracellular polysaccharides (EPS) [^4^]. Biofilm formation is an intricate process in which cells switch from planktonic growth mode to the sessile one. It is a multi-step process triggering specific mechanisms in bacteria. The mechanisms contributing to genetic and physiological heterogeneity involve genotypic variation caused by mutation and selection and adaptation to local environmental conditions, leading to differences in gene regulation. According to estimates, over 99% of bacteria in natural environments exist as biofilms rather than in planktonic cells. In such a community, bacteria are much more resistant to adverse environmental factors and antimicrobials [^5^]. Although *C.* jejuni seems to be a poor biofilm initiator and forms monospecies biofilm under specific growth conditions, it can survive in the environment by forming mixed biofilms with other microorganisms [^6^]. *Campylobacter* biofilms are present in many natural niches, including poultry houses, slaughterhouses, and numerous aquatic environments. As a result, *Campylobacter* might be transmitted from the environment to and within poultry farms, potentially contributing to pathogenesis in humans [^7,8^]. The molecular basis underlying biofilm formation by *Campylobacter* lags behind that of *Pseudomonas aeruginosa* or *Escherichia coli.* The first stage of biofilm formation in *C. jejuni* is flagellum-mediated, whereas extracellular DNA and DNA-binding proteins are essential for biofilm maturation [^9^]. Already identified motility-associated genes affecting biofilm formation in *C.* jejuni include genes encoding flagellins *flaA*, *flaB*, the filament cap *fliD*, the basal body *flgG*, *flgG2*, cell adhesion *flaC*, alternative sigma factor *fliA*, putative flagellar gene *fliS*, regulated by *flhA* [^10,11^] and Campylobacter bile resistance regulator *cbrR* [^12^]. Genes involved in quorum sensing, e.g., *luxS* encoding autoinducer-2, chemotaxis, e.g., *cheA*, *cheY*, *cheW*, and *cheV* as well as cell surface modification, including the *waaF* heptosyltransferase, the *lgtF* LPS biosynthesis glycosyltransferase, the *pglB* oligosaccharyltransferase, *peb4* antigenic virulence factor and *pgp1* required for peptidoglycan modification were also found to be essential for biofilm formation [^13–18^]. Stress response genes also play a critical role in *C. jejuni* biofilm formation, including *ahpC* (alkyl hydroxide reductase), *katA* (catalase A), *perR* (peroxide stress response regulator), *cosR* (Campylobacter oxidative stress regulator), *csrA* (carbon starvation regulator), *spoT* (stringent response regulator), *cprRS* (Campylobacter planktonic growth regulator), *ppk1–2* (polyphosphate kinase1, 2), and *phoX* (alkaline phosphatase) [^19–24^]. However, biofilm formation is a complex process, and there have not been saturating screens for genes involved in biofilm synthesis. One approach allowing the identification of potential biofilm-associated genes is a random transposon mutagenesis. Random transposon libraries are usually produced using mariner-based transposons or Tn5-based vectors. Tn5 vectors are active in a wide range of bacterial species. Tn5 transposons have been used to study biofilm process in *E. coli*, *P. putida*, *S. epidermidis*, *L. monocytogenes* and *C. jejuni* [^25–29^].

The present study aimed to study the role of the *cydB* gene, identified using random transposon mutagenesis, in biofilm formation by *C. jejuni*.

## Materials and Methods

### Bacterial Strains and Growth Conditions

The study was conducted on *C. jejuni* 81–176 [^30^]. The strain was stored at −80 °C in 15% glycerol. Prior to the experiments, *C. jejuni* 81–176 was subcultivated on Mueller-Hinton (MH) agar for 24 h at 42 °C under microaerobic conditions (85% N_2_, 5% O_2_, 10% CO_2_). The strains were then plated onto fresh MH agar supplemented with 20 μg/ml cefoperazone, 10 μg/ml vancomycin, 2 μg/ml amphotericin B and incubated under the same conditions. Liquid cultures of *C. jejuni* were grown in MH broth (MHB) and cultured in microaerobic environment.

### Electrocompetent C. jejuni cells

*C. jejuni* 81–176 cells for electroporation were prepared as described previously (^21^). Briefly, bacteria of OD_600_ 0.35–0.4 were incubated on ice for 30 minutes and were centrifuged at 4°C. The resulting pellet was washed with sterile cold distilled water and three times with ice-cold 10% glycerol and finally resuspended in 40 μl of GYT medium (10% glycerol, 0.125% yeast extract, 0.25% tryptone).

### Generation of transposon library

Random transposon mutagenesis was performed using the EZ-Tn5^™^ <KAN-2> Insertion Kit (Lucigen). Competent *C. jejuni* 81–176 cells were electroporated with 1 μl of transposome. Electroporation was performed in Bio-Rad Gene Pulser Xcell (2.5kV, 25 μF, 200 Ω). Following electroporation, the cells were resuspended in 100 μl of MHB, transferred on blood MH agar and allowed to recover for 6 h at 42°C in a microaerobic atmosphere. MH agar with kanamycin (30 μg/ml) was used to select the transformants.

### Identification of transposon-interrupted genes

Genomic DNA from mutants was extracted using Master Pure DNA Purification Kit (Lucigen). Transposon-interrupted genes were determined by sequencing flanking DNA following amplification of the region using the arbitrary primer PCR [^31^]. The resulting product was visualized by electrophoresis and single bands were sent for sequencing.

### Biofilm assay

Single colonies of bacteria were grown in MHB for 2 days. Next, bacteria were diluted to OD_600_ = 0.2 and incubated statically for 72 hours at 42° C in a microaerobic atmosphere in 96-well plates. The crystal violet (CV) method described by Fields and Thompson (2008) was used to assess biofilm formation. Briefly, bacterial suspension was aspirated, wells were washed with water, stained with CV, rinsed 3 times with water. The biofilm was dissolved in 80% DMSO and absorbance was measured at 570 nm. At least three experiments in triplicate were conducted for each strain.

### Construction of a ΔcydB mutant

The generation of the Δ*cydB* mutant of *C. jejuni* 81–176 strain was based on streptomycin counterselection system developed by Rathbun et al. [^32^]. First, the *cydB* gene was amplified using the cydB-F and cydB-R primers, and the resulting product was cloned into the pCR II-TOPO vector. The resulting plasmid pJK101 was then subjected to inverse PCR using primers cydB- invF and cydB-invR. The amplified product was digested (AgeI/DpnI ) and autoligated generating a new plasmid pJK102. Primers rpsLcat-F and rpsLcat-R were used to amplify the *rpsLcat* cassette (Str^S^, Cm^R^) from pKR021[^32^](Tab. 1). The pJK101 plasmid and *rpsLcat* cassette were cut with restriction enzymes (AgeI/NheI ) and ligated create pJK103. Next, a spontaneous derivative of streptomycin-resistant *C. jejuni* strain 81–176 was electroporated with the mutant allele and plated on MH agar with chloramphenicol (20 μg/ml). The antibiotic resistance cassette was replaced by electroporation of transformants (Cm^R^ Str^S^) with the pJK102 plasmid and selection on MH agar with 500, 1000, 2000 μg/ml streptomycin. Successful deletion of the *cydB* gene was confirmed by screening the mutant with PCR primers cydBspr- F and cydBspr-R.

### Complementation of ΔcydB mutant

To ensure that the observed phenotype of the mutant strain is specific to the deleted region of interest, complementation of the mutant with the wild copy of the gene was performed. Briefly, the *cydB* gene was amplified with primers cydB-compF and cydB-compR and inserted into the vector pRY112 (Cm^R^) (kindly provided by dr Hendrixson). The resulting plasmid CM- 1 was then introduced into *E. coli* DH5α/pRK212.1 donor strain (kindly provided by dr Hendrixson). The plasmid was then introduced into 81–176 *C. jejuni* mutant strain by conjugation. Transconjugates were recovered on MH agar with streptomycin (100 μg/ml), chloramphenicol (10 μg/ml ) and trimethoprim (10 μg/ml). The isolated CM-1 plasmid was sent for sequencing.

### Biofilm Analysis Using Scanning Electron Microscopy (SEM)

The biofilm for SEM analysis was prepared using the adsorption-incubation method described by Krzyżek et al. [^33^]. Briefly, 2 ml of bacterial suspension (OD_600_ 0.2) in MHB was added into a 6-well plate containing MHA and incubated for 3 days. The supernatant was gently removed, and the agar fragments were rinsed with PBS to eliminate loosely adhered bacterial cells. Agar fragments with attached biofilms were fixed with 2.5% glutaraldehyde, washed three times in 0.1 M cacodylate buffer, treated with increasing ethanol concentration gradient, sputtered with a carbon layer and observed with a Scanning Electron Microscope Auriga 60 (Oberkochen, Germany). For each strain two independent experiments were performed.

### Biofilm Analysis Using Confocal Laser Scanning Microscopy (CLSM)

The biofilm for CLSM was prepared as described Bronnec et al., [^34^]. Briefly, 1 ml of bacterial suspension (OD_600_ 0.5) in MHB was added into a 24-well plate and incubated for 2 hours at 42°C (85% N2, 5% O2, 10% CO2). Next, the supernatant was replaced by fresh MH broth and the incubation was continued for 3 days. Bacterial cells were stained with SYTO9 and PI according to manufacturer’s protocol (LIVE/DEAD kit, Thermo Fisher, Carlsbad, CA, USA) and washed with PBS. The biofilms were imaged as described by [^35^]. The SYTO 9-labeled living cells were detected using 488 nm lasers (Zeiss/Leica) and 502–538 nm emission range. PI-labeled dead or apoptotic cells were visualized with 561 nm (Zeiss) or 552 nm (Leica) lasers and 575–625 nm emission range. Biofilm-containing areas within a well of a 96-multiwell plate were imaged as 3×3 or 4×6 mosaics with a 7 μm interval in Z axis. The images were thresholded based on a set intensity value and the biofilm area was quantified using FIJI/ImageJ’s Analyze Particles function in relation to the well area (expressed as a percentage). Two independent experiments were performed for each strain.

### Biofilm formation in a Microfluidic System

Adhesion and biofilm formation in continuous flow was analyzed using microfluidic Bioflux 1000z system (Fluxion Biosciences, Alamenda, USA) according to protocol described by Paluch et al. (2021) with minor modifications. One milliliter of bacterial suspension (10^8^ CFU/ml) was placed in the 48-well plate and incubated for 1 minute. Next, the flow initiated at 0.2 dyn per 1 cm ^2^ was initiated [^36^]. The plate was incubated for 48 h at 42°C (85% N2, 5% O2, 10% CO2). The analysis of images were performed using Image J software. For each strain three independent experiments in triplicate were conducted.

### Motility test

To assess motility, a single colony was grown in MHB until OD_600_ = 0.2. Then the inoculum (1μl) was stabbed using 10 μl pipette into the middle of a 9 -cm petri dish containing 25 ml of 0.4 % agar MH and incubated overnight at 42° C in a microaerobic atmosphere (^32^).

### Growth curve

Overnight cultures of *C. jejuni* were harvested and diluted in MHB to OD_600_ = 0.05 and incubated at 42 °C under a microaerobic atmosphere. Samples were collected every 3 hours and the OD_600_ values were measured to compare the growth of studied strains.

### Statistical analysis

All statistical analyses were performed using Statistica 13.1. Each experiment was repeated at least three times. To assess the significance of the observed differences, one-way ANOVA and Tukey post-hoc test were used for Ez-Tn5 mutants. ANOVA Kruskal-Wallis with Bonferroni correction was used to determine whether differences in biofilm formation between the *cydB* mutant, the wild type, and the complemented strain are significant. A one-way ANOVA was used to evaluate whether differences in the motility were significant, whereas a multivariate ANOVA was used for viability and growth curves. Significance was set at a level of p < 0.05.

## Results

### Transposon mutagenesis of C. jejuni

**Fig. 1.**
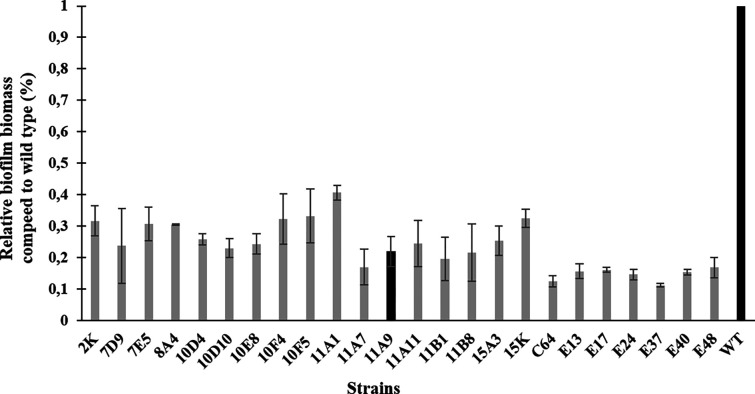
Biofilm formation of *C. jejuni 81–176* Tn5 mutants based on CV staining of cells after 72 h of growth. The data show mean absorbance relative to the wild type strain. Error bars show the standard deviations from at least three independent experiments. Statistically significant results were considered when p-value < 0.05. Black bars show the *cydB* gene mutant and wild type strain.

To identify genes that are involved in biofilm formation, a transposon library of *C. jejuni* 81–176 was constructed and the biofilm production ability of individual mutants compared to the wild-type strain was assessed. Nearly 1350 mutants were generated. Twenty-four mutants displayed a significant decrease in biofilm formation compared to wild type (2.46- to 8.84-fold) (Fig. 1). Biofilm-compromised mutants contained interruptions in genes related to motility, metabolism, glycosylation, membrane transport, and respiration. We identified new genes not previously linked to biofilm formation in *C. jejuni*, including the *cydB* encoding cytochrome d ubiquinol oxidase (Tab. 2). Since most genes encoded hypothetical or putative proteins, further study was focused on the confirmation of the *cydB* role in the biofilm formation process.

### Effect of cydB deletion on biofilm formation

**Fig. 3.**
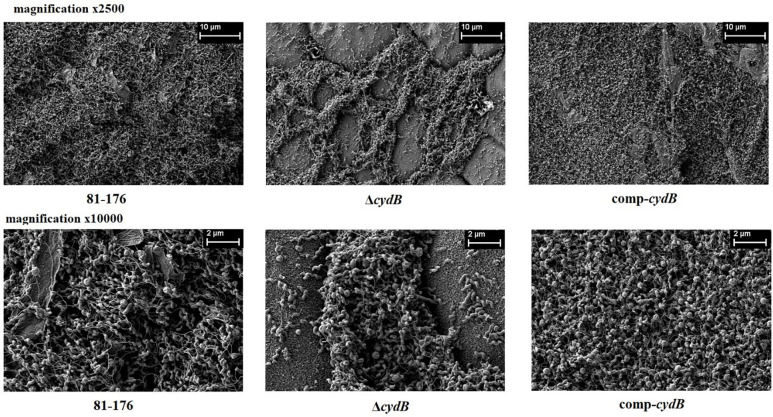
Biofilm microstructure observed by scanning electron microscopy at 72 h. SEM results show that the Δ*cydB* mutant strains had a lower biofilm volume and irregular structure (magnification × 2,500 and ×10,000).

To determine the role of *the cydB* gene in biofilm formation the biofilm formation abilities of *C. jejuni* 81–176 wild type, Δ*cydB,* and comp-*cydB* were compared. The Δ*cydB* mutant had significantly (p<0.05) reduced capacity to produce biofilm than either the wild type strain or complemented mutant (Fig. 2A). The OD_570_ measurements for the wild type, the mutant and the complemented mutant were 0.46 ± 0.09, 0.18 ± 0.06 and 0.48 ± 0.11, respectively. The Δ*cydB* mutant formed a very weak biofilm of loose structure on the bottoms of the wells (Fig. 2B). On the contrary, the biofilm of the wild type and the complemented strain was dense. To confirm the CV staining data, SEM was used to visualize the biofilm structure. In addition, the biofilm under dynamic conditions and cell viability were assessed. SEM revealed three-dimensional *C. jejuni* biofilm architecture that correlated with CV staining. The wild-type C. jejuni 81–176 strain and comp-*cydB* strain formed very dense mature biofilm consisting of huge agglomerates. These biofilms were practically indistinguishable. In contrast, the Δ*cydB* mutant formed a loosely organized biofilm of a much smaller volume and irregular structure (Fig 3). Only in some areas cell aggregates were visible, whereas void spaces with single cells dominated. The Δ*cydB* biofilm appeared more compact and less dense, as compared to the more uniform biofilm lawns of the wild-type and comp-*cydB* strains. All studied biofilms contained two cell morphology types, i.e., typical spiral shape and coccoidal shape, indicating the VBNC state. In addition, tubular criss-crossed network-like structures, probably corresponding to flagella, were visible (Fig. 3).

**Fig. 4.**
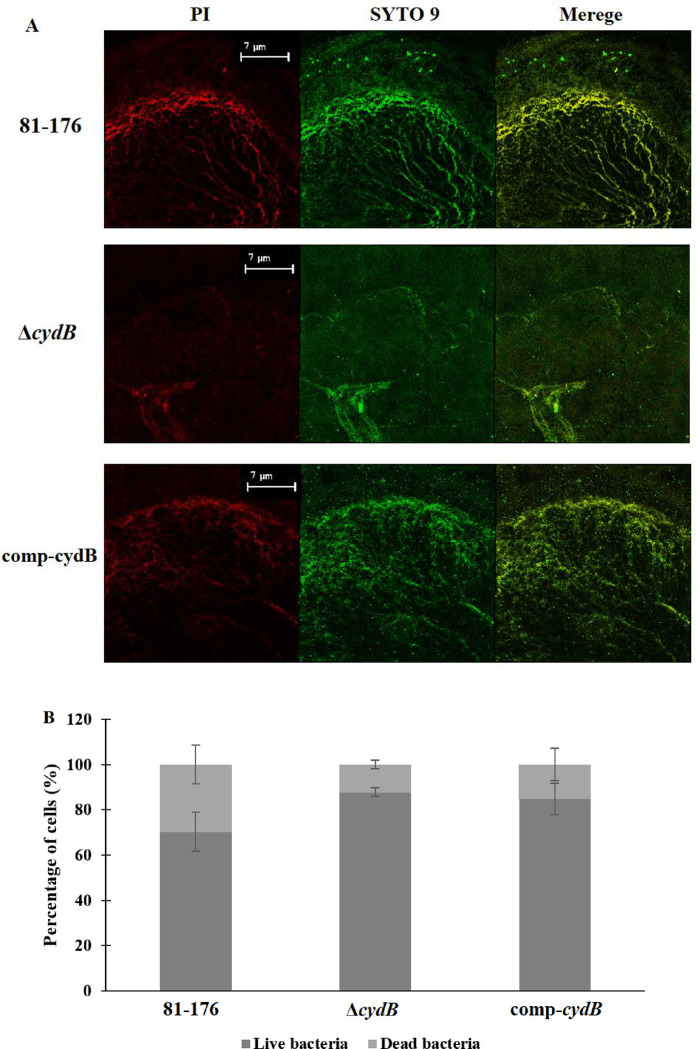
Representative CLSM image of live (SYTO 9), dead (PI) and merged bacterial cells in *C. jejuni* biofilms (A). Percentage of live (green) and dead cells (red) calculated from three randomly selected images. Data are presented as mean ± SD (B).

CLSM images allowed estimation of live and dead cells within the biofilm (Fig. 4A, 4B). The biofilm of the parent strain consisted of 70,3% ± 8,58% of viable cells (green) and 29,7% ± 8,58% of dead cells (red). The Δ*cydB* mutant biofilm contained 87,77% ± 1,81% of viable cells and 12,23% ± 1,81% of dead cells. The percentage of live and dead cells in the biofilm of comp-*cydB* strain was 84,75% ± 7,05% and 15,25% ± 7,05%, respectively. The differences in viability between studied strains were not significant (Fig. 4B).

**Fig. 5.**
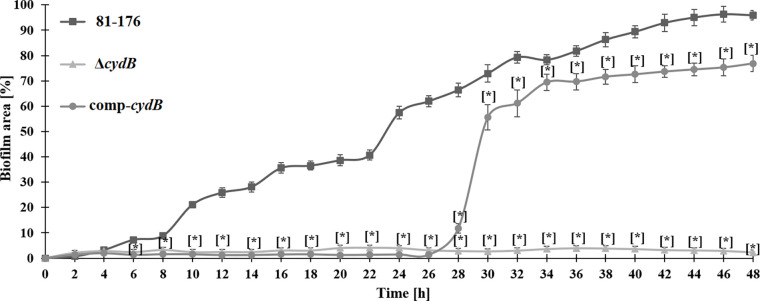
Dynamics of biofilm formation in the microfluidic channel (BioFlux 1000z) for *Campylobacter jejuni* strains during 48 h of incubation under flow conditions (0.2 dynes/cm2); mean ± SD, n = 3; * statistically different from control p < 0.05.

The Microfluidic Bioflux system allowed biofilm assessment under flow conditions (Fig. 5). For the wild-type strain systematic and gradual increase in the biofilm surface was noted. After 22 h biofilm reached over 50% of the microfluidic channel. Then further systematic increase in the biofilm surface was observed, reaching over 90% of the channel after 48 h. In the case of the Δ*cydB* mutant, the biofilm formation was very limited during the whole experiment, reaching only 2–4% of the channel surface. In contrast, the complemented strain increased biofilm formation after 26 h. Between 28 h and 30 h, the biofilm surface expanded five times. Then systematic increase in the biofilm surface was found, reaching over 75% of the channel.

### *Effect of cydB deletion on* the growth and motility of *C. jejuni*

**Fig. 6.**
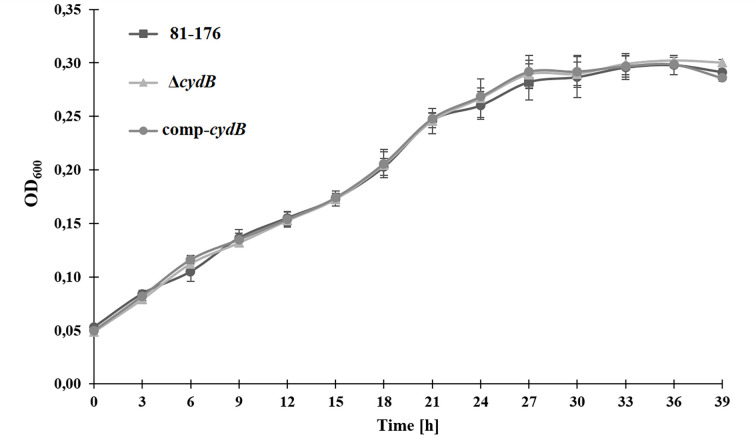
Growth curves of *C. jejuni* strains. *C. jejuni* 81–176 WT, Δ*cydB*, and comp-*cydB* strains were initially adjusted to OD_600_ of 0.05, and then cultivated in MH broth under microaerobic conditions at 42 °C for 39 h.

**Fig. 7.**
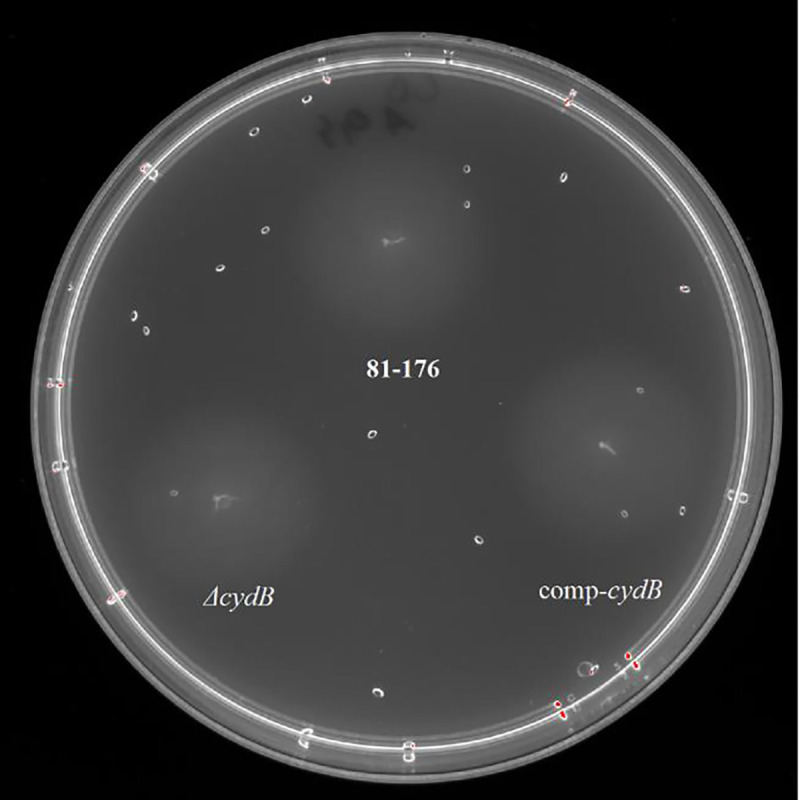
Motility of *C. jejuni* strains after 24-h incubation. Bacterial suspension (OD_600_ = 0.2) was stabbed using 10 μl pipette into the middle of 0.4 % MH agar plate and incubated overnight at 42° C in a microaerobic atmosphere.

The deletion of the *cydB* gene did not affect *C. jejuni* growth. There were no significant differences (p>0.05) in the OD_600_ values between the mutant, the parent strain, and the complemented mutant at all time points (Fig. 6). There were also no significant differences (p>0.05) in the motility between the mutant, the wild-type strain, and the complemented mutant after 24 hours (Fig. 7). The growth zones for the mutant, the parent strain, and the complemented mutant were 20.5 ± 1.38, 21.92 ± 1.44, and 21.33 ± 1.3, respectively.

## Discussion

*C. jejuni* is the major cause of bacterial, watery diarrhea in humans worldwide. Due to specific growth requirements and vulnerability, the actual number of *C. jejuni* infections might be underestimated. *Campylobacter* seems to have also unique molecular mechanisms underlying its pathogenesis, persistence, and survival [^2^]. In contrast to other enteropathogens, *C. jejuni* is naturally competent for DNA transformation and thereby may easily take up foreign DNA, including antibiotic resistance genes [^37^]. A substantial role in the survival and persistence in the environment plays biofilm formation. Since in the biofilm the pathogen transfers and acquires antibiotic-resistance genes more often than in planktonic cells, it is a relevant reservoir of antibiotic-resistant bacteria and a serious threat to public health [^8^]. For this reason, studying the mechanism helping survive *C. jejuni* in the environment, including biofilm formation, is of great importance. Around thirty genes responsible for the regulation and dynamics of *C. jejuni* biofilm formation have been described [^38^]. Studies on *P. aeruginosa* or *E. coli* have shown that this process is multifactorial, orchestrated by the expression of many genes belonging to various metabolic pathways. In *P. aeruginosa*, a kinetic model of the metabolic network on genome scale revealed 239 reactions whose inhibition resulted in either a decrease or increase in biofilm formation [^39^]. In *E. coli* Niba et al. [^40^] identified 110 genes which knockout resulted in reduced biofilm formation. More, microarray studies revealed 2504 differentially regulated genes in the mature biofilm of *P. aeruginosa* [^41^] and 1292 differentially expressed genes in the biofilm of *E. coli* cells [^42^]. A recent study by Tram et al. [^18^], using a comparative omics approach, has revealed distinct variations between the biofilm and planktonic state of *C. jejuni*. The transcriptome analysis showed 620 genes regulated expressed in biofilm conditions, confirming the complexity of the biofilm formation process in *C. jejuni* [^18^]. To identify new genes linked with biofilm formation we used a commercial transposon mutagenesis system, i.e., the EZ-Tn5 Transposome. Tn5 originated as bacterial transposons and has no target sequence requirement for insertion, potentially allowing higher insertion density than the mariner transposition system [^43^]. Lin et al. [^44^] have demonstrated that Tn5 transposon is an efficient tool for the systematic characterization of functionally relevant genes in *C. jejuni*. Mandal et al. [^45^] have used Tn5 technology in *Campylobacter jejuni* for essential genome studies. In turn, Teh et al. [^28^] applying this method, have screened biofilm-associated genes in *C. jejuni*. The authors have generated only 22 mutants on one out of 7 *C. jejuni* strains, suggesting a strain-dependent transposon efficiency [^28^]. In the present study, we constructed the library of over one thousand *C. jejuni* 81–176 mutants, which were assessed for biofilm formation ability compared to the parent strain. Twenty-four mutants displayed a significant decrease (2.46- to 8.84-fold) in biofilm formation compared to the wild type. Some mutants contained insertions in genes previously reported to affect biofilm formation, such as motility-associated genes *flgG*, *pflA* [^9,11^] or the *pglB* gene involved in glycosylation [^14^].This supports the reliability of EZ-Tn5 transposon mutagenesis. We have identified genes related to cell adhesion, metabolism, membrane transport, and respiration that were not previously linked with the biofilm formation in *Campylobacter*. The majority of these genes encode hypothetical proteins whose role in *C. jejuni* is not fully recognized or was not studied at all. The deletion of one gene, CJJ8176_1389, significantly decreased the colonization of chickens’ gastrointestinal tracts [^46^]. In the present paper, we focused on the impact of the *cydB* gene on biofilm formation by *C. jejuni.* This gene is associated with the respiratory chain and metabolism. In *C. jejuni* the *cydB* gene is located in the *cydAB* operon. In *E. coli* the *cydABX* gene cluster encodes cytochrome bd, a high-affinity quinol oxidase responsible for aerobic respiration in low-oxygen environments [^47^].The authors have shown that the loss of cytochrome bd affected biofilm architecture. The *cydAB* genes knockout reduced the abundance of extracellular matrix and increased bacterial sensitivity to nitrosative and oxidative stress [^47^]. Further, Beebout et al. [^48^] have revealed that the cytochrome bd loss also decreased biofilm resistance to antibiotics, indicating it as a possible target in the antibiofilm approach. In *C. jejuni cydAB* genes encode a cyanide-insensitive, low-affinity oxidase that was found to improve the survival of *C. jejuni* under microaerobic conditions [^49^]. Jackson et al. have demonstrated that this oxidase is not relevant for *C. jejuni* growth but affects the cell viability in a microaerophilic atmosphere. The authors have suggested that this oxidase should be renamed CioAB (cyanide-insensitive oxidase) due to a lack of characteristic cytochrome *bd* features [^49^]. To confirm the role of this gene, we constructed a non-marked deletion mutant together with complementation. The mutation did not affect the motility, bacterial growth, and viability of cells that could influence the biofilm formation. We found that the deletion of the *cydB* gene significantly decreased biofilm formation ability. The mutant produced biofilm of loosely organized structure and much lower volume than the parent strain. The complementation restored the parental phenotype, proving that the observed effect is attributed exclusively to the *cydB* knockout. Microfluidic Bioflux system allowed us to investigate the dynamic of biofilm formation. For the parent strain and complemented strain, systematic and gradual increase in the biofilm surface was observed. The complemented strain triggered biofilm production later than the wild-type strain which can be explained by the necessary adjustment to environmental conditions and overdue expression of the *cydB* gene located on the plasmid. On the contrary, the biofilm formation by the knockout-mutant strain was very scarce during the whole experiment, reaching only 2–4% of the microfluidic channel surface. In the current study, we demonstrated for the first time the role of the *cydB* gene in the biofilm formation process in *C. jejuni*. We also showed that the EZ-Tn5 system is a reliable and effective tool for studying the biofilm formation mechanism in *C. jejuni* 81–176.

## Conclusions

*C. jejuni* is one of the most important human pathogens. Despite its vulnerability to environmental stress, campylobacteriosis is the most prevalent zoonosis in the EU for over a decade. The biofilm lifestyle has been postulated to be a key factor contributing to the high prevalence of *C. jejuni* and its ability to overcome environmental stress. Since the mechanism underlying biofilm formation in *C. jejuni* is still not well recognized, there is a need for detailed research. In our study we showed that the EZ-Tn5 Transposome system is an efficient tool for exploring the molecular basis of biofilm formation. We identified a new gene, c*ydB*, involved in biofilm formation by *C. jejuni*. By more fully understanding the mechanisms of *C. jejuni* biofilm formation, we hope to be able to rationally design biofilm-disrupting inhibitors.

## Figures and Tables

**Fig. 2A.**
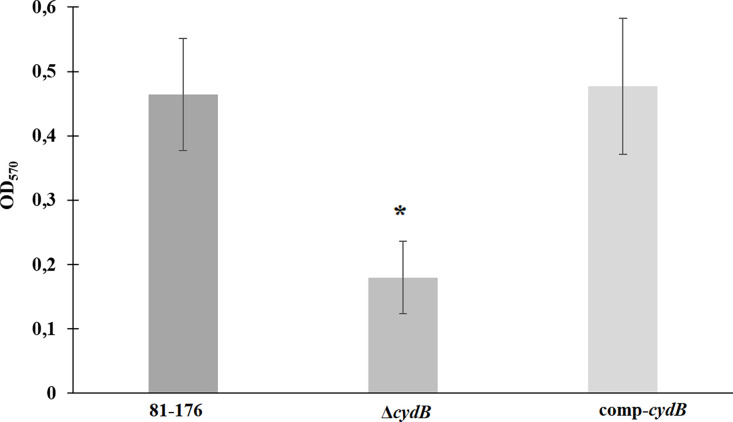
Biofilm formation of *C. jejuni* strains. Bacterial suspensions (OD_600_ = 0.2) of 81–176 WT, Δ*cydB*, and comp-*cydB* strains were incubated statically for 72 h at 42°C, and stained with CV. Then OD_570_ was measured to quantify biofilm formation. An asterisk represents statistical significance (P<0.05).

**Fig. 2B.**
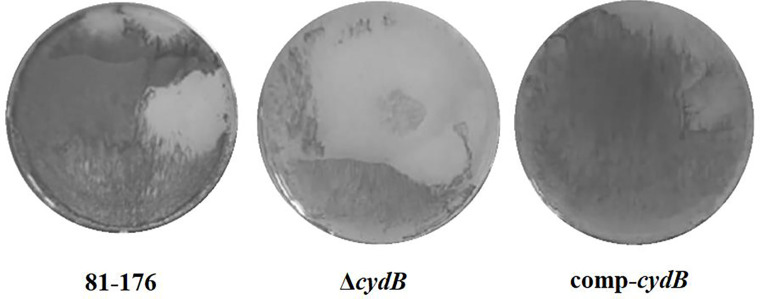
Biofilms on plate after 72-h incubation based on CV staining.

**Tab. 1:** List of Primers Used in This Study

Primer	Sequence

cydB-F	5’ GCTATGGCAAATCACAAATCC 3’
cydB-R	5’ CTAAATACACAATGACTCAGG 3’
cydBinv-F	5’**ACCGGT**TAT**GCTAGC**GTAAAAATCACACGCGAAG 3’
cydBinv-R	5’**ACCGGT**ACTTAAAATCAACCACCAAT 3’
rpsLcat-F	5’ ACCGGTAACGACTAAAGTTTTAACA 3’
rpsLcat-R	5’ **GCTAGC**TTATTTATTCAGCAAGTCTT 3’
cydBspr-F	5’ GAACCTATGCAAAATCGTATCG 3’
cydBspr-R	5’ GCAAAAAGCTCTGCTAAGG 3’
cydB-compF	5’CGC**GGATCC**tttatgatatagtggatagatttatgatataatgagttatcaacaaatcggaatttacggaggataaatgTTTTTTGGTTTAGAACTTGAAGG 3’
cydB-compR	5’ CGC**CTGCAG**TTAATATGCGTGATCATCGTTTG 3’

Bold capitals show the sequence of restriction enzyme sites, while underline shows the Pcat promoter sequence.

**Tab. 2:** Identification of transposon insertion sites in *C. jejuni* 81–176 mutants with decreased biofilm formation.

Mutant	Disrupted gene	Description	References
**2K**	CJJ81176_1389	hypothetical protein I	
**7D9**	CJJ8176_0721	*flgG*, flagellar base-body rod protein FlgG	^11]^
**7E5**	CJJ8176_1037	High affinity branched-chain amino acid ABC transporter, periplasmic amino acid-binding protein	
**8A4**	CJJ8176_0446	ABC transporter, ATP-binding protein	
**10D4**	CJJ8176_1312	conserved hypothetical protein	
**10D10**	CJJ8176_1435	Putative sugar transferase	
**10E8**	CJJ8176_1550	Paralyzed flagella protein PflA	
**10F4**	CJJ8176_0080	*flgD*, flagellar hook assembly protein	^9^
**10F5**	CJJ8176_0261	Putative membrane protein, matches to protein family HMM PFO1027	
**11A1**	CJJ81176_1187	Hypothetical protein TIGR01033	
**11A7**	CJJ8176_0430	Putative lipoprotein	
**11A9**	CJJ8176_0111	*cydB*, cytochrome d ubiquinol oxidase subunit II	
**11A11**	CJJ8176_1443	Hypothetical protein	
**11B1**	CJ18176_1143	*pglB*, general glycosylation pathway protein	^14^
**11B8**	CJJ8176_1525	Tungstate ABC transporter, periplasmic tungstatebinding protein, putative	
**15A3**	CJJ81176_1185	Conserved hypothetical protein	
**15K**	CJJ81176_0128	Hypothetical protein	
**C64**	CJJ81176_1245	Sodium/hydrogen exchanger family protein	
**E13**	CJJ81176_0034	Hypothetical protein	
**E17**	CJJ81176_1363	Hypothetical protein	
**E24**	CJJ81176_1495	Bifunctional putA protein, putative	
**E37**	CJJ81176_0728	Conserved hypothetical protein TIGR00486	
**E40**	CJJ81176_1309	Deoxycytidine triphosphate deaminase, putative	
**E48**	CJJ81176_1340	Motility accessory factor	

## Data Availability

The datasets analysed during the current study are available in the Wroclaw University of Environmental and Life Sciences repository (DOI:10.57755/dkrh-9228).
